# Computational Phenotyping in Psychiatry: A Worked Example

**DOI:** 10.1523/ENEURO.0049-16.2016

**Published:** 2016-08-02

**Authors:** Philipp Schwartenbeck, Karl Friston

**Affiliations:** 1 The Wellcome Trust Centre for Neuroimaging, UCL, London WC1N 3BG, UK; 2 Centre for Cognitive Neuroscience, University of Salzburg, 5020 Salzburg, Austria; 3 Neuroscience Institute, Christian-Doppler-Klinik, Paracelsus Medical University Salzburg, A-5020 Salzburg, Austria; 4 Max Planck UCL Centre for Computational Psychiatry and Ageing Research, London WC1B 5EH, UK

**Keywords:** active inference, computational psychiatry, generative model, Markov decision process, model inversion

## Abstract

Computational psychiatry is a rapidly emerging field that uses model-based quantities to infer the behavioral and neuronal abnormalities that underlie psychopathology. If successful, this approach promises key insights into (pathological) brain function as well as a more mechanistic and quantitative approach to psychiatric nosology—structuring therapeutic interventions and predicting response and relapse. The basic procedure in computational psychiatry is to build a computational model that formalizes a behavioral or neuronal process. Measured behavioral (or neuronal) responses are then used to infer the model parameters of a single subject or a group of subjects. Here, we provide an illustrative overview over this process, starting from the modeling of choice behavior in a specific task, simulating data, and then inverting that model to estimate group effects. Finally, we illustrate cross-validation to assess whether between-subject variables (e.g., diagnosis) can be recovered successfully. Our worked example uses a simple two-step maze task and a model of choice behavior based on (active) inference and Markov decision processes. The procedural steps and routines we illustrate are not restricted to a specific field of research or particular computational model but can, in principle, be applied in many domains of computational psychiatry.

## Significance Statement

We provide an overview over the process of using formal models to understand psychiatric conditions, which is central in the emerging research field of “computational psychiatry.” This approach promises key insights into both healthy and pathological brain function as well as a more mechanistic understanding of psychiatric nosology, which may have important consequences for therapeutic interventions or predicting response and relapse. In a worked example, we discuss the generic aspects of using a computational model to formalize a task, simulating data and estimating parameters, as well as inferring group effects between patients and healthy control subjects. We also provide routines that can be used for these steps and are freely available in the academic software SPM.

## Introduction

Recent advances in computational neuroscience—and the lack of a mechanistic classification system in mental disorders (Stephan et al., 2015)—have motivated the application of computational models in clinical research. This has led to the emergence of a field of research called computational psychiatry, which has attracted much recent interest (Friston et al., 2014b; Stephan and Mathys, 2014; Wang and Krystal, 2014; Huys et al., 2015). Its aim is to use computational models of behavior or neuronal function to infer the hidden causes of measurable quantities, such as symptoms, signs, and neuroimaging or psychophysical responses. In consequence, this approach promises new insights into the computational mechanisms of certain pathologies, which would otherwise be hidden, when assessing the observations alone.

**Figure 1.**
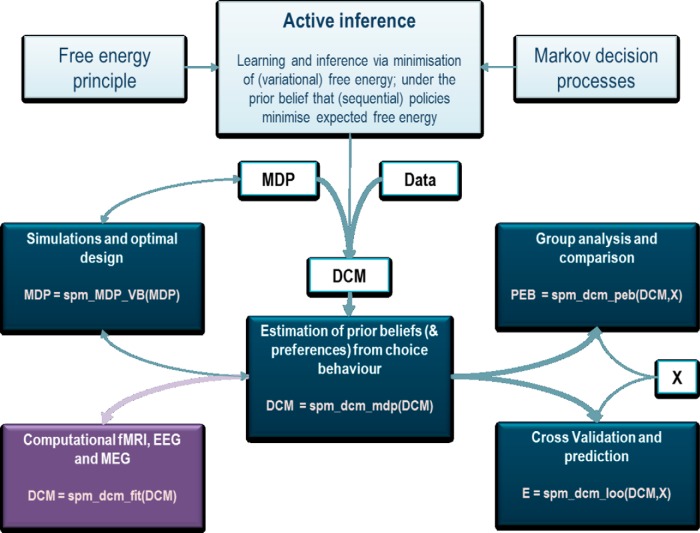
A schematic overview of the analysis stream underlying the treatment of computational psychiatry in this article. The basic procedure involves specifying a model of behavior cast in terms of a Markov decision process (MDP). Under the assumption that choices are made in an approximately Bayes optimal fashion using active (Bayesian) inference, this model is sufficient to predict behavior. If we supplement the model specification (MDP) with empirical choice behavior (Data), we can estimate the prior beliefs responsible for those choices. If this is repeated for a series of subjects, the ensuing priors can then be analyzed in a random-effects (PEB) model to make inferences about group effects or to perform cross-validation. Furthermore, physiological and behavioral predictions can be used as expansion variables for fMRI or other neuroimaging time series (bottom left). The routines in the boxes refer to MATLAB routines that are available in the academic software SPM. These routines are sufficient to both simulate behavioral responses and analyze empirical or observed choice behaviour, at both the within-subject and between-subject levels. The final routine also enables cross-validation and predictions about a new subject’s prior beliefs using a leave-one-out scheme that may be useful for establishing the predictive validity of any models that are considered.

This tutorial addresses a particular but important aspect of computational psychiatry; namely, how to characterize individuals in terms of their computational phenotypes. In other words, it describes how to quantify the beliefs and preferences of an individual—within a formal framework—by fitting their choice behavior to a computational model. Our focus is on a particular sequence of analyses that is supported by routines in the SPM software. These routines have been written in a way that they should be applicable to any choice or decision tasks that can be modeled in terms of (partially observable) Markov decision processes (Toussaint et al., 2006; Alagoz et al., 2010; Rao, 2010; FitzGerald et al., 2015; see below). The purpose of this note is to describe the overall structure of the analyses and the functionality of a few key (Matlab) routines that can be used to analyze the behavior of subjects in a relatively straightforward and efficient fashion. An overview of these routines is provided in Figure 1 and the software notes below.

Characterizing choice behavior in terms of a formal model represents a subtle challenge, especially under active inference models of behavior (Friston et al., 2011, 2015a). This is because it is generally assumed that subjects make decisions based on a generative model of the task at hand and behave in an approximately Bayesian way by making bounded rational choices (Berkes et al., 2011). Generative models provide a probabilistic mapping from hidden states or parameters to observations. In other words, a generative model specifies how consequences (i.e., observed outcomes) are generated from their causes (i.e., unobserved or hidden states and parameters). When modeling behavior under a generative model, the (objective) model includes the (subjective) model we assume is used by each subject (we provide a worked example of this in the following sections). This means that fitting choice behavior becomes a meta-Bayesian problem, in which we are trying to infer the beliefs adopted by a subject based on our beliefs about this (active) inference process (Daunizeau et al., 2010). Therefore, under the perspective of (active) Bayesian inference, the only things one can infer about a subject are their prior beliefs (Houlsby et al., 2013). In other schemes, for example normative economic models or reinforcement learning, prior expectations would correspond to key model parameters, such as temporal discounting or the sensitivity to rewards. In turn, this means that the difference between one subject and another has to be cast in terms of their generative models, which can always be formulated as prior beliefs or, when their priors pertain to the parameters of probability distributions, hyperpriors. Crucially, understanding individual behavior in terms of individual (subjective) generative models of a task adds an additional hypothesis space for investigating individual and group differences, and speaks to the idea of understanding pathological behavior in terms of pathological models of (i.e., abnormal prior beliefs about) the world (Beck et al., 2012; Dayan, 2014; Schwartenbeck et al., 2015c). In what follows, we will illustrate this general principle using a particular example (and simulated data). The end point of this analysis will be a characterization of single subjects (and group differences) in terms of (hyper) priors encoding uncertainty or confidence about choice behavior. However, the same procedure can be applied to any prior belief that shapes an individual’s response to their changing world.

## The formal approach

Formally, this approach rests on (both subjective and objective) generative models. For the subjective generative model, these observations are experimental outcomes or cues observed by a subject, while for the objective model, the outcomes would be the subject’s responses or choices. In statistical terms, generative models provide a likelihood function, P(y|Θ,m), of data y given a set of parameters Θ and the model structure m, as well as a prior over parameters, P(Θ|m). Crucially, one can invert this model using Bayes rule to infer the most likely parameter values (hidden states) causing observed data, as follows:

$$\frac{P(y|\Theta ,m),P(\Theta |m)}{P(y|m)}=P(\Theta |y,m).$$

Here, P(y|m) refers to the evidence or marginal likelihood of the model, which can be obtained by integrating or marginalizing out Θ in the numerator, as follows:

P(y|m)=∫P(y|Θ,m)⋅ P(Θ|m)dΘ.

Usually, this integral cannot be solved analytically (exactly) but has to be approximated, for example through heuristics like the Akaike/Bayesian information criterion or more refined but computationally more expensive solutions, such as sampling or variational methods (Attias, 2000; Beal, 2003; Bishop, 2006).

In the following, we will illustrate the use of generative models in computational phenotyping and focus on the crucial steps in (1) specifying the model, (2) using the model to simulate or generate data, (3) model inversion or fitting to estimate subject-specific parameters, and (4) subsequent inference about between-subject or group effects using hierarchical or empirical Bayes: for example, comparing a group of healthy control subjects to a patient group. We will use a simple decision-making task to illustrate these processes and use a recently proposed computational model of behavior, which casts decision-making as a Markov decision process based on (active) Bayesian inference (Friston et al., 2013; Friston et al., 2015). The details of the task and computational model are not of central importance and are discussed elsewhere (Friston et al., 2015a). Here, the model serves to illustrate the typical procedures in computational approaches to psychiatry. When appropriate, we will refer explicitly to Matlab routines that implement each step. These routines use (variational) procedures for Bayesian model inversion and comparison. They have been developed over decades as part of the SPM software, and have been applied extensively in the modeling of neuroimaging and other data. The key routines called on in this article are described in the software notes below.

## Model specification

Figure 1 provides an overview of the procedures we will be illustrating. Usually, one starts by developing and optimizing the task paradigm. Clearly, to describe a subject's response formally, it is necessary to specify a subjective generative model that can predict a subject’s responses. For experimental paradigms, it is often convenient to use discrete state–space models (i.e., partially observable Markov decision processes), in which various cues and choices can be labeled as discrete outcomes. We will illustrate this sort of model using a relatively simple two-step maze task.

### An active inference model of epistemic foraging

In the following, we introduce the ingredients for casting a decision-making or planning task as an active Bayesian inference. Note that this treatment serves as an illustration for the general steps in computational phenotyping, which do not depend on the use of a particular computational or normative model. In practice, one often wants to compare different variants of computational models of choice behavior, such as (Bayesian) inference and (reinforcement) learning models (Sutton and Barto, 1998), which we discuss below. In fact, the active inference routine we present here allows one to perform these formal comparisons, which are the subject of current research (Friston et al., 2009; Mathys et al., 2011; Schwartenbeck et al., 2015b).

**Figure 2.**
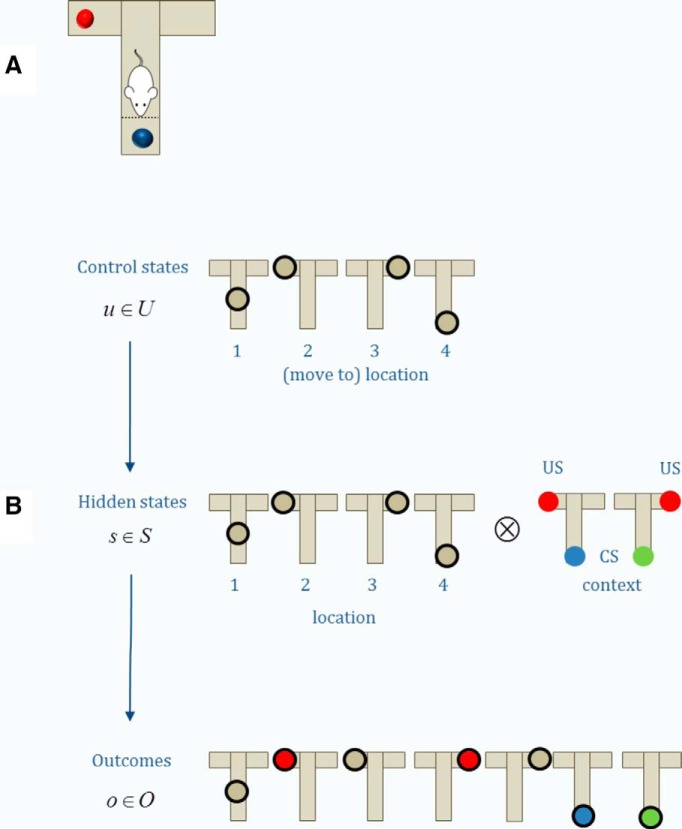
***A***, Task: we used a simple two-step maze task for our simulations, where a subject starts in the middle of a T-shaped maze and has to decide whether to sample the left or right arm, knowing that one of the two arms will contain a reward but it can sample only one of them (i.e., the arms are absorbing states). Alternatively, the subject could sample a cue at the bottom of the maze, which will tell her which arm to sample. ***B***, State space: Here, the subject has four different control states or actions available: she can move to the middle location, the left or the right arm or the cue location. Based on these control states, we can specify the hidden states, which are all possible states that a subject can visit in a task and often are only partially observable. In this task, the hidden state comprises the location × the context (reward left or right), resulting in 4 × 2 = 8 different hidden states. Finally, we have to specify the possible outcomes or observations that an agent can make. Here, the subject can find itself in the middle location, in the left or right arm with or without obtaining a reward or at the cue location.

For our worked example, we will use a task that requires both exploratory (epistemic) and exploitative behavior (Friston et al., 2015a). In brief, in this task a subject has to choose whether to sample the left or right arm of a T-shaped maze to obtain a reward or a cue. The rewards are in the upper arms, while the cue is in the lower arm. This cue indicates the location of the reward with high validity (Fig. 2A). Crucially, the left and the right arm of the maze are absorbing states, which means that the agent has to stick with its choice. Therefore, in the absence of any prior knowledge, the optimal policy (i.e., a sequence of actions) involves first sampling the cue and then selecting the reward location indicated by the cue. While this seems like a very simple task, it captures interesting aspects of behavior such as planning and a trade-off between exploration (i.e., sampling the cue) and exploitation (i.e., moving to the arm that is likely to contain a reward). As such, it can be easily extended to model more complex types of choice problems.

The first step is to specify the (subjective) generative model of this task. These sorts of problems can be modeled efficiently as partially observable Markov decision processes, where the transition probabilities are determined by the current action and state, but not by the history of previous states (see below). In the context of active inference, the specification of the generative model for a Markov decision process is based on three matrices, called *A*, *B*, and *C*. These describe the mapping from hidden states to outcomes, the transition probabilities and the preferences (expectations) over outcomes, respectively. The specification of these matrices depends on the state space of the task (Fig. 2B). In our example, the agent can be in one of eight possible hidden states that are determined by the agent’s location (middle, bottom, left arm, or right arm) and context (cue indicates the left or right arm).

**Figure 3.**
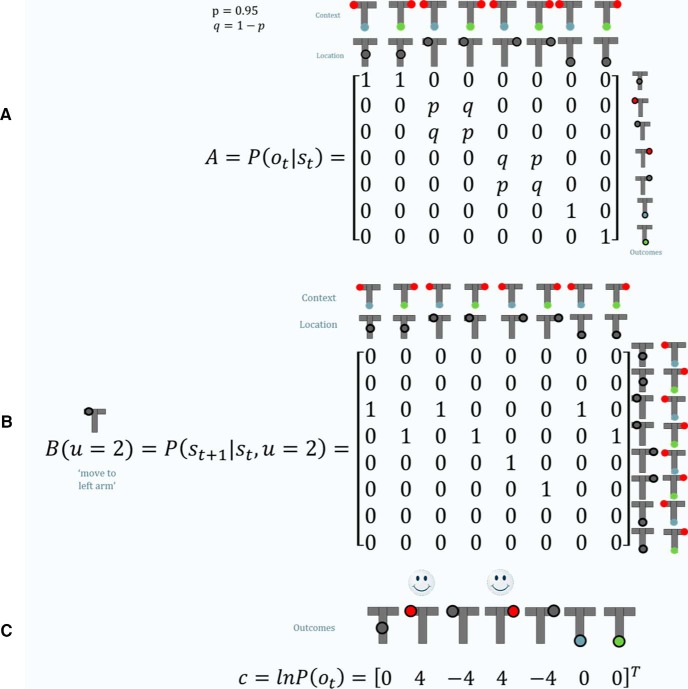
Generative model. ***A***, The *A*-matrix maps from hidden states to observable outcome states (resulting in a 7 × 8 matrix). There is a deterministic mapping when the subject is either in the middle position (simply observing that she is in the middle) or at the cue location (simply observing that she is at the cue location where the cue indicates either left or right). However, when the subject is in the left or right arm, there is a probabilistic mapping to a rewarded and an unrewarded outcome. For example, if the subject is in the left arm and the cue indicated is in the left arm (third column), there is a high probability, *p*, of a reward, whereas there is a low probability *q* = 1 − *p* of no reward. ***B***, The *B*-matrix encodes the transition probabilities (i.e. the mapping from the current hidden state to the next hidden state contingent on the action taken by the agent). Thus, we need as many *B*-matrices as there are actions available (four in this example). Illustrated here is the *B*-matrix for a move to the left arm. We see that the action never changes the context, but (deterministically) does change the location, by always bringing it to the left arm, except when starting from an absorbing state (right arm). ***C***, Finally, we have to specify the preferences over outcome states in a *C*-vector. Here, the subject strongly prefers ending up in a reward state and strongly dislikes ending up in a left or right arm with no reward, whereas it is somewhat indifferent about the “intermediate” states. Note that these preferences are (prior) beliefs or expectations; for example, the agent beliefs that a rewarding state is exp⁡(4)≈55 times more likely than an “intermediate” state [exp(0) = 1].

As the name implies, hidden states are usually not fully (but only partially) observable and have to be inferred based on observations. This motivates the *A*-matrix, which maps from hidden states to observations (outcome states; i.e., states that the subject can observe). Here, we can differentiate seven different observable states (middle position, left arm rewarded or unrewarded, right arm rewarded or unrewarded, cue location indicating to go left or right; Fig. 2B); thus, the *A*-matrix is a 7 × 8 matrix (Fig. 3A). The *A*-matrix accounts for any partially observable aspect of a Markov decision process, where this mapping becomes an identity matrix if all states are fully observable (i.e., if there is no uncertainty about which hidden state caused an observation).

Second, the *B*-matrix encodes the transition probabilities in a decision process (i.e. the probability of the next hidden state contingent on the current hidden state and the action taken by the agent; Fig. 3B, illustration of the transition probabilities in our task). These transition probabilities are a particular feature of Markov decision processes, because they depend only on the current state (and action), not on previous states. This is called the Markov or “memory-less” property. Finally, one has to specify the agent’s preferences over outcomes states (observations), which are encoded in the *C*-vector. Preferences over outcomes are (prior) expectations, which can be based on task instructions or, in the context of economic decision-making or reinforcement learning, utility or reward (or any combination of these).

From the perspective of active inference, the preferred (desired) states are the states that the subject expects to find itself in. Note that casting an agent’s preferences in terms of prior expectations does not imply that the concept of reward is meaningless; rather, the notion of a reward is absorbed into an agent’s expectations, which guide its inferences about policies. Therefore, a Bayes optimal subject will infer or select policies that bring about expected or preferred states. This inference is modeled by Bayesian updates that accumulate evidence to optimize posterior beliefs about hidden states of the world and the current policy being enacted. Mathematically, this can be expressed as a minimization of variational free energy (as an upper bound on surprise; see below). Because variational free energy is an approximation to negative Bayesian model evidence, belief updating to minimize free energy for surprise is exactly the same as maximizing model evidence. This Bayes optimal inference ensures that subjects obtain the outcomes they expect (i.e., desire). Equivalently, they will avoid unexpected (i.e., undesired) outcomes because they are surprising. The key quantities that endow behavior with a purposeful, goal-directed aspect are the prior preferences that nuance the selection of policies. These preferences are generally treated as free parameters that can be estimated through model inversion, as we will see below.

For a full discussion of the task described above and the specifics of casting choice behavior as an active inferential Markov decision process, please see (Friston et al., 2013; Friston et al., 2015a). Having said this, the particular details of this paradigm are not terribly important. The important thing to note is that, in principle, nearly every experimental paradigm can be specified with two sets of matrices (*A* and *B*), while every subject can be characterized in terms of their preferences (*C*). Practically speaking, the key challenge is not to specify these matrices; but, the greatest challenge is to understand and define the hidden state space implicit in the paradigm (in other words, the states in which these matrices operate).

## Simulating data

Having specified the subjective generative model for this task, we can now use the model to simulate or generate choice behavior. While eventually one will use real data, simulating data is useful to assess whether the generative model of a task produces sensible behavior. Once any counterintuitive behavior has been resolved, one can then use simulations to optimize the design parameters (or state space) that will be used empirically as the subjective model.

To simulate choices, we need to specify specific values for the priors and hyperpriors of the generative model, which we want to recover when working with real data (see Model inversion, below). In our case, we need to specify the following two parameters: the preferences (expectations) over outcomes and a hyperprior on the confidence or precision of beliefs about policies. The preferences over outcomes simply determine the desirability of each observable (outcome) state. For our simulations, we have assigned a high value to outcomes in which the agent obtains a reward (+4), a low value for outcome states in which the agent does not obtain a reward and is trapped in an absorbing state (−4), and a medium value for the remaining outcomes, after which it is still possible to obtain a reward later (0; Fig. 3C). Because these expectations are defined in log-space, this can be understood as the subject’s (prior) belief that obtaining a reward is exp(4)≈55 times more likely than ending up in a “neutral” state (exp(0) = 1). In addition, we can specify a hyperprior on precision. Precision (γ)

reflects an agent’s stochasticity or goal directedness in choice behavior but, crucially, itself has a Bayes-optimal solution that can be inferred on a trial-by-trial basis. The importance of precision in decision processes and its putative neuronal implementation are discussed in detail elsewhere (Friston et al., 2014a; Schwartenbeck et al., 2015a). In brief, the values of policies are scored as variational free energies, which follow a Gibbs distribution. Precision is the inverse temperature of this distribution and itself is parameterized by a γ distribution with a scale (α) and rate (β) hyperparameter. Thus, precision plays the same role as an inverse temperature in classic softmax choice rules, with the difference that it is continuously optimized. For our simulations, we have set α and β to a value of 2, resulting in an expected value for (inverse) precision of γ=1. Hyperpriors on precision are of great importance when recovering the parameters based on observed behavior and may play a central role in psychiatry, as discussed below.

Finally, we need to specify initial states for each trial in the experiment (i.e., the state from which the subject starts navigating through the maze). In our simulations, the initial (hidden) state of every trial is set to the middle location and (randomly) to one of the two contexts (reward on the left or right), where the entire experiment comprises 128 trials. Details of the model specification and simulation (and the steps below) can be found in the DEM toolbox of the academic software SPM12 (Wellcome Trust Centre for Neuroimaging, London, UK, http://www.fil.ion.ucl.ac.uk/spm) under the option “behavioral modeling.”

**Figure 4.**
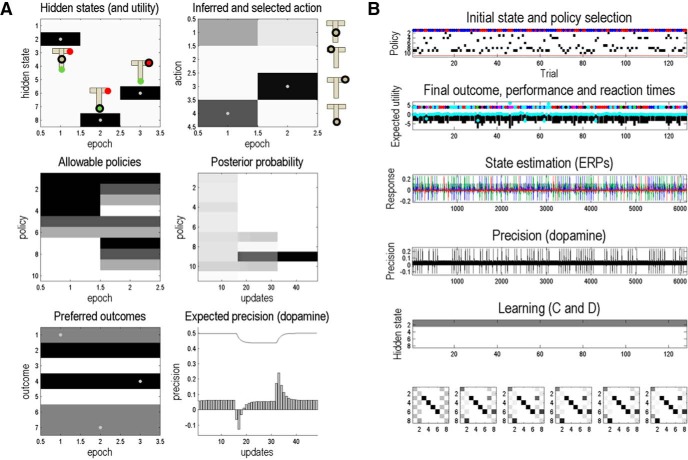
Data simulation using the routine spm_MDP_VB. ***A***, A simulated example trial, where the left top panel shows the hidden states, the right top panel shows the inferred actions, the middle panels show the inference on policies (i.e., the possible sequences of actions), the bottom left panel shows the preferences over outcome states (*c*-vector), and the bottom right panel shows the expected precision, which could be encoded by dopamine (Friston et al., n.d.). In this trial, the subject starts in the middle position where the reward is (most likely) on the right arm. She then makes a selection to sample the cue and, finally, moves to the right arm, as indicated by the cue (darker colors reflect higher posterior probabilities). ***B***, Overview of a simulated experiment comprising 128 trials. The first panel shows the inferred policies (black regions) and initial states (shown as colored circles: red circles, reward is located at the right arm; blue circles, reward is located at the left arm) at every given trial. The second panel shows estimated reaction times (cyan dots), outcome states (colored circles), and the value of those outcomes (black bars). Note that the value of outcomes is expressed in terms of an agent’s (expected) utility, which is defined as the logarithm of an agent’s prior expectations. Thus, the utility of an outcome is at most 0 [= log(1)]. Reaction times reflect the choice conflict at any given trial and are simulated by using the time it takes Matlab to simulate inference and subsequent choice in any given trial (using the tic-toc function in Matlab). The third and fourth panels show simulated event-related potentials for hidden state estimation and expected precision, respectively. The specifics of these simulations are discussed in detail elsewhere (Friston et al., 2016). Finally, panels five and six illustrate learning and habit formation. Our simulations did not include any learning or habitual responses.

Having specified the generative model, we can now use the function spm_MDP_VB to simulate behavior. This routine provides solutions of behavior based on active inference, such that agents believe they will minimize expected free energy. Expected free energy can be decomposed into the Kullback–Leibler (KL) divergence between predicted and preferred outcomes plus the expected surprise (uncertainty) about future outcomes. Therefore, minimizing expected free energy implicitly maximizes expected utility or preferred outcomes in a risk-sensitive fashion (compare with KL control), while resolving ambiguity. This active inference scheme is based on the following three variational update equations: agents are assumed to perform within-trial inference on current states, actions, and (expected) precision. In addition to these updates, the parameters of the generative model are updated between trials (i.e., learned). Details of the model and variational Bayesian updates can be found in the study by Friston et al. (2015a), and the output of these simulations can be found in Figure 4A for a single trial and in Figure 4B for the entire experiment.

These simulated responses (and other electrophysiological responses not considered in this article) can now be used to verify the efficiency of the paradigm and its basic behavior. One can also assess the sensitivity of simulated behavior to variations in preferences and prior precision. The sensitivity determines the efficiency with which a subject’s preferences and hyperpriors can be estimated. We now turn to this estimation in terms of model inversion.

## Model inversion

We have described the first two steps of computational modeling; namely, translating a particular paradigm into a generative model and simulating data by exploiting the ability of generative models to simulate or generate data. In these simulations, we used a specific model that casts decision-making as a Markov decision process based on active inference. While recent work has highlighted the role of deficient decision processes in psychiatry (Montague et al., 2012; Hauser et al., 2014; Huys et al., 2015), the central prerequisite of defining a generative model for a task generalizes to all applications in computational psychiatry.

We can now turn to the inversion of the generative model to recover its parameters, based on observed (or in our case simulated) behavior. This is an important step in empirical research, because the aim of computational psychiatry is to characterize psychopathology in a quantitative and computationally meaningful fashion. In other words, we want to explain people's behavior in terms of a specific parameterization of a (subjective) generative model.

A common approach is to compute maximum *a posteriori* (MAP) estimates of parameters obtained by inverting an objective generative model. As described above, a generative model is a mapping from hidden parameters to observed data. Thus, by inverting the model one can map from observations to hidden parameters, resulting in a posterior distribution over the most likely (MAP) parameters contingent on the model and observed data. To do so, one has to decide on how to approximate the model evidence or marginal likelihood. Here, we will use (negative) variational free energy as a proxy for the log-evidence of a model. The log-model evidence can be expressed as follows:

$$In\;P(y|\;m)\;=\;D_{KL}[q(\Theta )||]P(\Theta |y,m)]+F(q(\Theta ),y),$$

where the first term is the KL divergence between the true posterior and an approximate posterior, which has a lower bound of zero, and thus makes the (negative) variational free energy in the second term a lower bound of the (negative) log-model evidence (which is at most zero). It is thus sufficient to minimize free energy to maximize the log-evidence of the model itself. When fitting subject-specific choices, the data at the observed choices and the active inference scheme cited above provide a likelihood model. The likelihood is the probability of obtaining a particular sequence of choices, given a subject’s preferences and hyperpriors. Model inversion corresponds to estimating the preferences and hyperpriors, given an observed sequence of choices.

To do this model inversion, one can use the routine spm_dcm_mdp, which inverts an objective generative model given the subjective model, observed states, and responses of a subject. This is made particularly easy because the subjective model provides the probability of various choices or actions from which the subject selects behavior. We can now simply integrate or solve the active inference scheme using the actual outcomes observed empirically and evaluate the probability of the ensuing choices. To complete the objective generative model, we only need to specify priors over the unknown model parameters. In what follows, we use fairly uninformative shrinkage priors. Shrinkage generally refers to any regularization method in statistics that prevents overfitting (but see Bishop, 2006, page 10). In particular, shrinkage is inherent in Bayesian inference due to the use of priors, which “shrink” the parameter estimates toward the prior mean, and thus preclude overfitting. In our case, we used priors with a mean of 0 and a variance of 1/16, thus inducing shrinkage toward 0. These priors can be changed in the spm_dcm_mdp.m script, to specify any prior constraints on, or knowledge about, the parameters that are estimated (e.g., time constants that fall in natural ranges). We will see later that priors can themselves be optimized, using Bayesian model comparison (BMC). This follows because any model is defined in terms of its (shrinkage) priors. Technically, model inversion uses a standard (Newton method) gradient ascent on variational free energy (in which the curvature or Hessian is estimated numerically). Practically, this involves specifying the (MDP) model used to explain the subject’s behavior, the observed outcomes, and their associated choices or actions (and objective priors on the unknown subjective model parameters). These quantities are specified as fields in a Matlab structure usually called DCM (for dynamic causal model).

**Figure 5.**
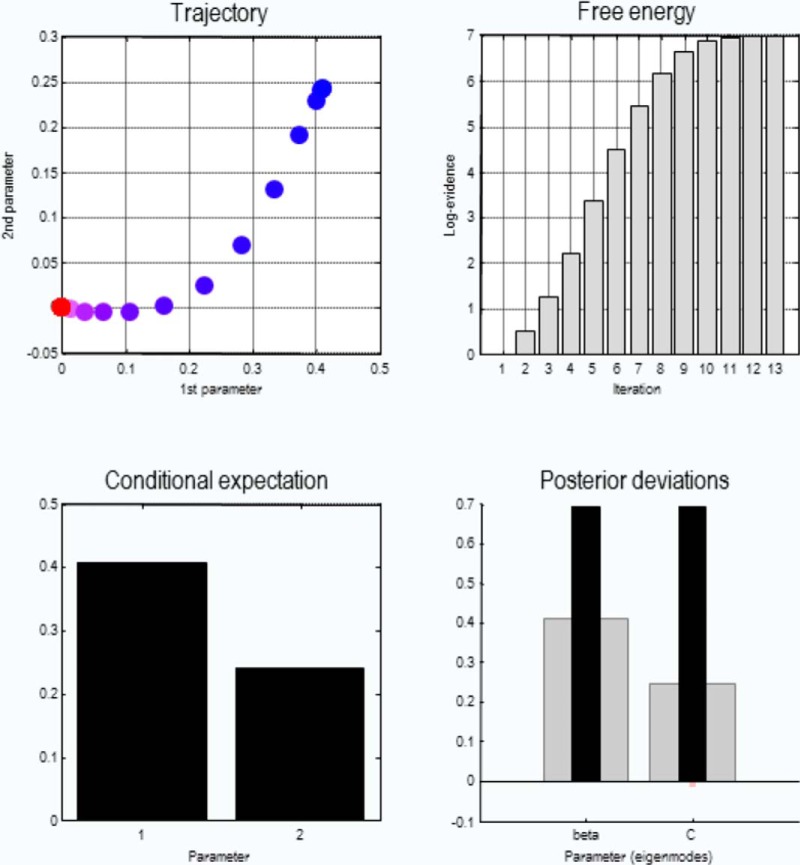
Model inversion, as implemented by the routine spm_dcm_mdp, is based on simulated behavior. In this routine, (negative) variational free energy as a lower bound of log-model evidence is maximized and converges after the 13th iteration (top right). The trajectory of two estimated parameters in parameter space is provided (top left) as well as their final conditional estimates (bottom left) and their posterior deviation from the prior value (bottom right). The black bars on the bottom right show the true values, while the gray bars show the conditional estimates, illustrating a characteristic shrinkage toward the prior mean.

**Figure 6.**
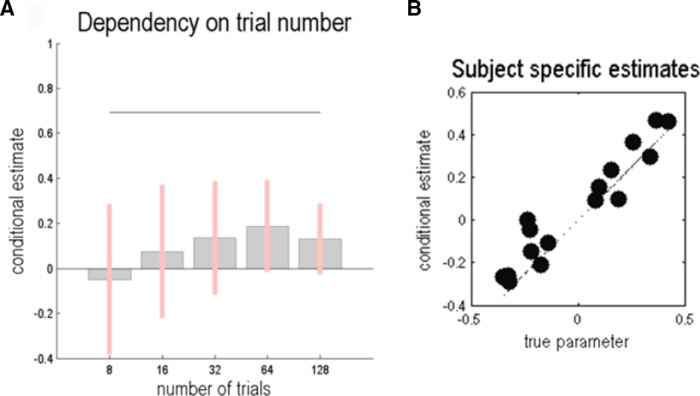
***A***, Conditional estimate and confidence interval for the hyperprior on precision (β) as a function of the number of trials in a simulated experiment. ***B***, True and estimated subject-specific parameters, following model inversion for 16 subjects with a group effect in the hyperprior (β). The two groups can be seen as two clusters along the diagonal.

Figure 5 shows the output of this routine when applied to our simulated data. This provides estimates of the preferences over outcomes and the hyperprior β on precision. Here, the estimation converges at the 13th iteration, and provides the trajectory of the two parameters as well as their conditional expectations and posterior deviations. The inversion of simulated data can also be helpful to ensure that the subjective model can be inverted prior to testing subjects or patients. For example, one can simulate how many trials are necessary to recover parameters with sufficient confidence. An example of this is shown in Figure 6A for the hyperprior on precision.

Importantly, there is usually more than one free parameter in a subject’s generative model, and these parameters could have similar effects on behavior. One can use intuitions about the effects of two parameters on response variables and use simulations to test for any similarity and any ensuing conditional dependencies. An efficient parameterization (or experimental design) would usually suppress conditional dependencies and therefore make the parameter estimation more efficient. In our example, we treated the hyperprior on precision and the preferences over outcomes as free parameters, where the former accounts for an agent’s stochasticity in behavior and the latter controls the agent’s preferences for different states. Thus, these two parameters control distinct aspects of observable behavior and can be estimated relatively efficiently. Any conditional dependencies among parameters can be assessed (*post hoc*) in terms of their shared variance; for example, by assessing their posterior covariance.

## Inferring group effects

Finally, having described the specification of the generative model and model inversion, we can now turn to inference about between-subject or group effects. As outlined above, when casting decision-making as a Markov decision process based on active inference, a key role is played by precision, which determines the confidence that subjects place in their beliefs about choices. Furthermore, it has been previously suggested that precision might be encoded by neuromodulators, in particular dopamine (Friston et al., 2012; Friston et al., 2014a; FitzGerald et al., 2015; Schwartenbeck et al., 2015a), but also acetylcholine (Moran et al., 2013). Therefore, precision might be central for understanding certain pathologies, such as psychosis, obsessive–compulsive disorder, or addiction (Adams et al., 2013; Schwartenbeck et al., 2015c; Hauser et al., 2016).

We simulated a group difference in precision by repeating the model specification, data simulation, and model inversion steps described above for two groups of eight subjects each; where, crucially, we introduced a difference in the hyperprior on precision (of one-quarter) between the two groups and an intersubject variability with a log precision of four (SD, 0.135). The result of the model inversion for each subject is illustrated in Figure 6B in terms of real and estimated subject-specific hyperpriors. These results immediately indicate of a group effect in this parameter that is further evidenced by a significant difference between the estimates in the two groups (t=6.627, p<0.001). Notice that inferences about group effects call on a model of between-subject differences. In Bayesian terms, these models are called hierarchical models or empirical Bayesian models and are the Bayesian equivalent of (random-effects) ANOVAs, with between-subject and within-subject effects. A full random-effects analysis can be implemented using parametric empirical Bayes (PEB) implemented for nonlinear models of the sort we are dealing with here (for details, see Efron and Morris, 1973; Friston et al., 2015b; Friston et al., 2015c).

The Matlab routine to directly assess the group effect is spm_dcm_peb. In brief, this routine uses hierarchical empirical Bayes for second-level group inversion based on a design matrix modeling group effects. In this case, the between-subject model (or design matrix) contained two explanatory variables modeling a group mean and a group difference, respectively. This is the between-subject model *X* in Figure 1 (for details, see Friston et al., 2015b,c; for a reproducibility study using this approach, see Litvak et al., 2015).

**Figure 7.**
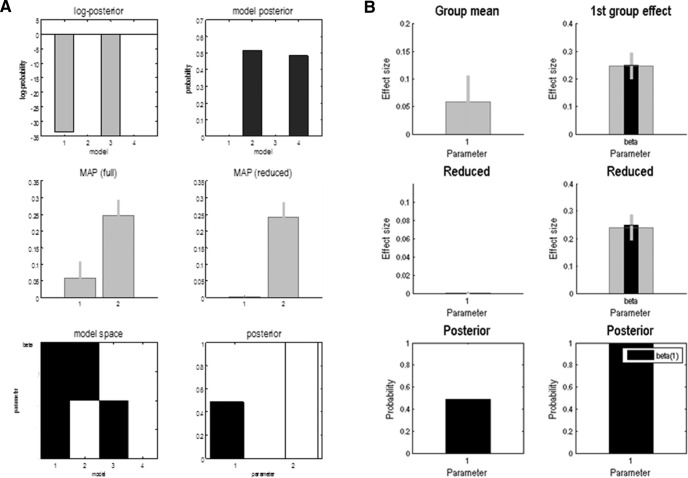
Hierarchical empirical Bayesian inference on group effects using the function spm_dcm_peb. ***A***, Results of Bayesian model comparison (reduction) to infer whether the full model (with both group mean and group differences) or a reduced (nested) model (bottom left) provides a better explanation for the data. These results indicate high posterior evidence for a model with a group difference, with slightly less evidence for the full model, which also includes a group mean effect (i.e., a deviation from the group prior mean; top panels). Middle panels show the maximum *a posteriori* estimates of the mean and group effects for the full and reduced models. ***B***, Estimated (gray bars) group mean (left) and difference (right) in β. These estimates are about one-quarter (top right), which corresponds to the group effect that was introduced in the simulations (black bars). The small bars correspond to 90% Bayesian confidence intervals. A reduced parameter estimate corresponds to the Bayesian model average over all possible models (full and reduced) following Bayesian model reduction.

Figure 7A shows the output of Bayesian model comparison, which can be understood as the posterior evidence that there is a group difference in the full model (i.e., a group mean and difference) or in a reduced model (i.e. only one or no effects). In Figure 7A (top right), we find that the models with a group mean and difference and models with just a group difference have the highest posterior evidence (with a posterior probability that is slightly < 0.5 and >0.5, respectively). Figure 7B shows the corresponding parameter estimates (the group mean results are shown on the left, and the group differences are shown on the right). Here, Figure 7B (top right) shows that the group difference of one-quarter is recovered accurately. Note that while these results correspond to the result obtained by a simple *t* test, using this routine for Bayesian model comparison offers more flexibility and provides more information than standard parametric approaches. One obvious difference, in relation to classic inference, is the possibility of assessing the posterior evidence for the null hypothesis (i.e., evidence that there is no difference between groups) and the posterior estimate of the effect size for the group difference. For example, if we repeat the above simulation with a group difference of zero, we obtain a high posterior probability for the model with no mean and group differences (≈0.7) and the models that assume a group difference scoring a posterior probability of <0.1, whereas a nonsignificant *t* test would only provide inconclusive results.

While this empirical Bayesian procedure is very useful for inferring group differences within a model, in practice, researchers are also interested in comparing different models of the same (choice response) data. For example, although we have illustrated computational phenotyping based on active inference, other models that are used in computational neuroscience (and psychiatry) are based on biophysical neural network models or reinforcement learning (for recent review, see Huys et al., 2016). Comparing different models of a task, such as inference and (reinforcement) learning models, is necessary to identify which computational framework best explains observed behavior. This usually rests on some form of Bayesian model comparison (Stephan et al., 2009). In the present setting, competing models are compared in terms of their model evidence, as scored by variational free energy computed during model inversion (at the within-subject level or at the between-subject level using spm_dcm_peb). This has been shown to outperform alternative approximations to model evidence, such as the Akaike and Bayesian information criteria (Penny, 2012). It is possible to perform such model comparisons within the active inference toolbox; for example, by comparing active inference (surprise minimization) to classic expected utility theory (Schwartenbeck et al., 2015a,b).

Furthermore, one might also be interested in formal model comparisons that entertain different hidden state spaces underlying the (subjective) generative models used by subjects. Crucially, patient groups and healthy control subjects might differ in both the hyperparameters of their subjective models (e.g., the hyperprior on precision) as well as the state space underlying these models. For example, we have illustrated a subjective model in which precision is a hidden state that has to be inferred. However, it is possible that some subjects or patient groups do not perform inference on precision, such that their best model would be one in which precision is not inferred. Likewise, patient groups and healthy control subjects might differ in how they represent prior expectations; for example, by encoding losses and wins separately. This can be tested by using formal model comparisons as described above, where models that include precision as a hidden state are compared to models where no inference on precision is made, or to models that have a different parameterization for losses and wins. This has been the focus of previous work (Schwartenbeck et al., 2015b) and, importantly, also speaks to the issue of Bayesian model comparison and averaging (FitzGerald et al., 2014), as well as structure learning (Tervo et al., 2016), when performing a task. More generally, any assumption that is implicit in the form of hyperparameterization of a model can be tested empirically through Bayesian model comparison. In this sense, the particular model used to illustrate the approach in this article should not be taken too seriously; every aspect can be optimized in relation to empirical choice behavior until an optimal description of the paradigm is identified.

**Figure 8.**
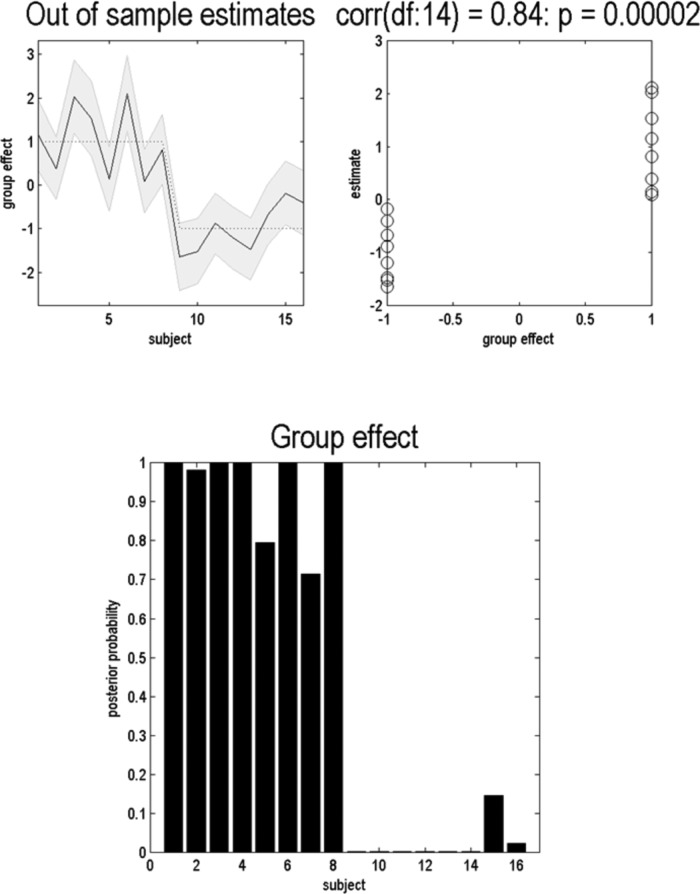
Cross-validation based on a leave-one-out scheme. Using the function spm_dcm_loo, we find that group membership is accurately recovered based on the parameter estimate of the hyperprior on each subject. This is evidenced by a high correlation between inferred and true group membership in the top right panel. These reflect out-of-sample estimates of effect sizes, which were large (by design) in this example. The top right panel provides the estimate of the group indicator variable (which is +1 for the first group and −1 for the second group). The bottom panel provides the posterior probability that each subject belongs to the first group.

**Figure 9.**
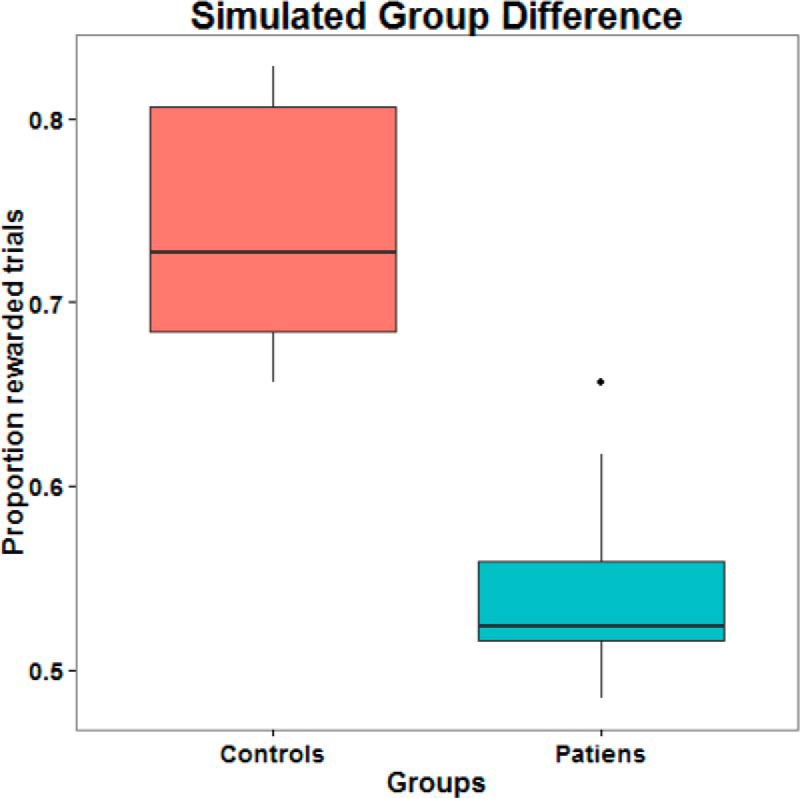
Simulated group difference between control subjects and patients (with a group difference in precision of one-quarter) in the average reward received. Note that this difference in an observable variable was successfully traced back to a difference in the hyperprior on precision (a latent variable) by our inference scheme, which is important because such inverse problems are usually ill posed and hard to solve.

Finally, we can use a cross-validation scheme implemented by the routine spm_dcm_loo to test whether group membership can be accurately recovered, based on a leave-one-out scheme. This scheme uses the predictive posterior density of a predictive variable (Friston et al., 2015c), which is β in our example. In brief, this cross-validation or predictive scheme switches the roles of the between-subject explanatory variables (e.g., diagnosis) and the subject-specific parameter estimates they are trying to explain. This allows one to classify a particular subject based on that subject’s parameter estimates, and to classify group effects based on independent data. Figure 8 shows the results of this cross-validation scheme, which speaks to a high accuracy in recovering the group membership, as indicated by a high correlation between recovered and actual group membership (Fig. 8, top right) and a conclusive posterior probability for group membership for each subject (Fig. 8, bottom). This facility may be useful for classifying new subjects based on their computational phenotyping. Note that the inferred difference in the latent variable precision (Fig. 7) and the inferred group membership based on this latent variable (Fig. 8) corresponds closely with the actual (simulated) group difference in an observed variable; namely, the average reward received by each group in this task (Fig. 9). Importantly, this shows that the inference scheme discussed above can infer the latent variable underlying the observed variables. This is important because the implicit inverse problem is usually ill posed and can have many solutions.

A common approach to evaluate the goodness of different models relies on cross-validation. In other words, model parameters are optimized using a (training) subset of the data and tested on another (test) subset. Model performance can then be assessed purely in terms of accuracy, without having to worry about complexity. This is because the use of independent training and test data precludes overfitting. Therefore, cross-validation accuracy can be regarded as a proxy for model evidence and used, in a straightforward way, to compare different models. The approaches described in this article eschew cross-validation, because the model evidence is assessed directly through its variational free-energy approximation. There are pros and cons of a variational assessment of model evidence, in relation to cross-validation accuracy. In brief, the variational approach is universally more efficient (by Neyman–Pearson Lemma) than cross-validation. This can be seen heuristically by noting that the model inversion and parameter estimation in cross-validation uses incomplete (training) data (e.g., a leave one scheme). On the other hand, cross-validation accuracy is robust to model assumptions, and does not rely on variational approximations to model evidence. This means that it can be useful in assessing the robustness of variational schemes of the sort described in this article.

## Conclusion

We have tried to provide an overview of the key steps entailed by phenotyping in computational psychiatry, using a worked example based on a (Markov) decision process. The first, and probably most important, step is the specification of the (subjective) generative model for a task or paradigm. This model encodes a mapping from hidden states or parameters to outcomes and can be used to simulate (generate) data. More importantly, it forms the basis of an objective generative model for empirical choice behavior; enabling one to map from choices to (subject-specific) model (hyper) parameters. This allows one to estimate the prior preferences and hyperpriors used by the subject to select their behavior.

Our simulations were based on a particular computational approach called active inference, which casts (choice) behavior and planning as pure Bayesian inference with respect to an agent’s prior expectations. This approach can be particularly useful when we want to cast a decision process as inference [i.e., assuming a stable (subjective) generative model that is used to infer hidden states or policies]. Furthermore, this allows one to compare a (Bayesian) inference model to a (Bayesian or reinforcement) learning model, in which the parameterization of the (subjective) generative model is continuously updated (FitzGerald et al., 2014). Future work will implement the aspect of learning within the active inference scheme, such that the parameterization of the generative model can be updated and simultaneously used to infer hidden states. A limitation of this computational toolbox is that it provides solutions only for discrete state-space problems, which significantly simplifies a given decision or planning problem at the expense of biological realism about the inferred neuronal mechanisms underlying the decision process.

An important aim of computational psychiatry is to characterize the generative processes that underlie pathological choice behavior. A first step to achieve this is to perform single-subject model inversion to estimate their prior beliefs and then compare these estimates at the between-subject level. Hierarchical or empirical Bayes and Bayesian cross-validation can then be used to test hypotheses about group differences such as diagnosis or response to treatment. While we have used a specific example of Markov decision processes based on active Bayesian inference, the procedures we have described are generic, and may provide an exciting, straightforward, and principled approach to personalized treatment in psychiatry.

## Software notes

Here we describe the key routines called on in the text. These routines are called in a demo script that can be edited and executed to change various parameters. The demo script is described first, followed by the key routines it calls.

### DEM_demo_MDP_fit

This routine uses a Markov decision process formulation of active inference (with variational Bayes) to model foraging for information in a three-arm maze. This demo illustrates the inversion of single-subject and group data to make inferences about subject-specific parameters, such as their prior beliefs about precision and utility. We first generate some synthetic data for a single subject and illustrate the recovery of key parameters using variational Laplace. We then consider the inversion of multiple trials from a group of subjects to illustrate the use of empirical Bayes in making inferences at the between-subject level. Finally, we demonstrate the use of Bayesian cross-validation to retrieve out-of-sample estimates (and the classification of new subjects).

In this example, the agent starts at the center of a three-way maze that is baited with a reward in one of the two upper arms. However, the rewarded arm changes from trial to trial. Crucially, the agent can identify where the reward (US) is located by accessing a cue (CS) in the lower arm. This tells the agent whether the reward is on the left or the right upper arm. This means the optimal policy would first involve maximizing information gain or epistemic value by moving to the lower arm and then claiming the reward thus signified. Here, there are eight hidden states (four locations times right or left reward), four control states (that take the agent to the four locations), and seven outcomes (three locations times two cues plus the center). The central location has an ambiguous or uninformative outcome, and the upper arms are rewarded probabilistically.

### spm_MDP_VB

*% active inference and learning using variational Bayes*

*% FORMAT [MDP] = spm_MDP_VB(MDP,OPTIONS)*

*%*

*% MDP.S(N,1)- true initial state*

*% MDP.V(T - 1,P)- P allowable policies (control sequences)*

*%*

*% MDP.A(O,N)- likelihood of O outcomes given N hidden states*

*% MDP.B{M}(N,N)- transition probabilities among hidden states (priors)*

*% MDP.C(N,1)- prior preferences (prior over future outcomes)*

*% MDP.D(N,1)- prior probabilities (prior over initial states)*

*%*

*% MDP.a(O,N)- concentration parameters for A*

*% MDP.b{M}(N,N)- concentration parameters for B*

*% MDP.c(N,N)- concentration parameters for habitual B*

*% MDP.d(N,1)- concentration parameters for D*

*% MDP.e(P,1)- concentration parameters for u*

*%*

*% optional:*

*% MDP.s(1,T)- vector of true states*

*% MDP.o(1,T)- vector of observations*

*% MDP.u(1,T)- vector of actions*

*% MDP.w(1,T)- vector of precisions*

*%*

*% MDP.alpha- upper bound on precision (Gamma hyperprior – shape [1])*

*% MDP.beta- precision over precision (Gamma hyperprior - rate [1])*

*%*

*% OPTIONS.plot- switch to suppress graphics: (default: [0])*

*% OPTIONS.scheme- {'Free Energy' | 'KL Control' | 'Expected Utility'};*

*% OPTIONS.habit- switch to suppress habit learning: (default: [1])*

*%*

*%*

*% produces:*

*%*

*% MDP.P(M,T)- probability of emitting action 1,…,M at time 1,…,T*

*% MDP.Q(N,T)- an array of conditional (posterior) expectations over*

*%N hidden states and time 1,…,T*

*% MDP.X- and Bayesian model averages over policies*

*% MDP.R- conditional expectations over policies*

*%*

*% MDP.un- simulated neuronal encoding of hidden states*

*% MDP.xn- simulated neuronal encoding of policies*

*% MDP.wn- simulated neuronal encoding of precision (tonic)*

*% MDP.dn- simulated dopamine responses (phasic)*

*% MDP.rt- simulated reaction times*

This routine provides solutions of an active inference scheme (minimization of variational free energy) using a generative model based on a Markov decision process. This model and inference scheme is formulated in discrete space and time. This means that the generative model and process are finite-state machines or hidden Markov models, whose dynamics are given by transition probabilities among states, and the likelihood corresponds to the probability of an outcome given hidden states. For simplicity, this routine assumes that action (the world) and hidden control states (in the model) are isomorphic.

This implementation equips agents with the prior beliefs that they will maximize expected free energy: expected free energy is the free energy of future outcomes under the posterior predictive distribution. This can be interpreted in several ways)—most intuitively as minimizing the KL divergence between predicted and preferred outcomes (specified as prior beliefs)—while simultaneously minimizing the (predicted) entropy of outcomes conditioned on hidden states. Expected free energy therefore combines KL optimality based on preferences or utility functions with epistemic value or information gain.

This particular scheme is designed for any allowable policies or control sequences specified in MDP.V. Constraints on allowable policies can limit the numerics or combinatorics considerably. For example, situations in which one action can be selected at one time can be reduced to T polices, with one (shift) control being emitted at all possible time points. This specification of polices simplifies the generative model, allowing a fairly exhaustive model of potential outcomes, eschewing a mean field approximation over successive control states. In brief, the agent encodes beliefs about hidden states in the past and in the future conditioned on each policy (and a nonsequential state–state policy called a habit). These conditional expectations are used to evaluate the (path integral) of free energy that then determines the prior over policies. This prior is used to create a predictive distribution over outcomes, which specifies the next action.

In addition to state estimation and policy selection, the scheme also updates model parameters, including the state transition matrices, mapping to outcomes, and the initial state. This is useful for learning the context. In addition, by observing its own behavior, the agent will automatically learn habits. Finally, by observing policies chosen over trials, the agent develops prior expectations or beliefs about what it will do. If these priors (over policies, which include the habit) render some policies unlikely (using an Ockham's window), they will not be evaluated.

### spm_dcm_mdp

*% MDP inversion using Variational Bayes*

*% FORMAT [DCM] = spm_dcm_mdp(DCM)*

*%*

*% Expects:*

*%———————————————————————*

*% DCM.MDP% MDP structure specifying a generative model*

*% DCM.field% parameter (field) names to optimize*

*% DCM.U% cell array of outcomes (stimuli)*

*% DCM.Y% cell array of responses (action)*

*%*

*% Returns:*

*%———————————————————————*

*% DCM.M% generative model (DCM)*

*% DCM.Ep% Conditional means (structure)*

*% DCM.Cp% Conditional covariances*

*% DCM.F% (negative) Free-energy bound on log evidence*

This routine inverts (cell arrays of) trials specified in terms of the stimuli or outcomes and subsequent choices or responses. It first computes the prior expectations (and covariances) of the free parameters specified by DCM.field. These parameters are log-scaling parameters that are applied to the fields of DCM.MDP.

If there is no learning implicit in multitrial games, only unique trials (as specified by the stimuli) are used to generate (subjective) posteriors over choice or action. Otherwise, all trials are used in the order specified. The ensuing posterior probabilities over choices are used with the specified choices or actions to evaluate their log probability. This is used to optimize the MDP (hyper) parameters in DCM.field using variational Laplace (with numerical evaluation of the curvature).

### spm_dcm_peb

*% Hierarchical (PEB) inversion of DCMs using BMR and VL*

*% FORMAT [PEB,DCM] = spm_dcm_peb(DCM,M,field)*

*% FORMAT [PEB,DCM] = spm_dcm_peb(DCM,X,field)*

*%*

*% DCM - {N [x M]} structure array of DCMs from N subjects*

*% ————————————————————————-*

*% DCM{i}.M.pE- prior expectation of parameters*

*% DCM{i}.M.pC- prior covariances of parameters*

*% DCM{i}.Ep- posterior expectations*

*% DCM{i}.Cp- posterior covariance*

*% DCM{i}.F- free energy*

*%*

*% M.X- second-level design matrix, where X(:,1) = ones(N,1) [default]*

*% M.pC- second-level prior covariances of parameters*

*% M.hE- second-level prior expectation of log precisions*

*% M.hC- second-level prior covariances of log precisions*

*% M.bE- third-level prior expectation of parameters*

*% M.bC- third-level prior covariances of parameters*

*%*

*% M.Q- covariance components: {'single','fields','all','none'}*

*% M.beta- within:between precision ratio: [default = 16]*

*%*

*% field- parameter fields in DCM{i}.Ep to optimize [default: {'A','B'}]*

*%'All' will invoke all fields. This argument effectively allows*

*%one to specify the parameters that constitute random effects.*

*%*

*% PEB- hierarchical dynamic model*

*% ————————————————————————-*

*% PEB.Snames- string array of first-level model names*

*% PEB.Pnames- string array of parameters of interest*

*% PEB.Pind- indices of parameters in spm_vec(DCM{i}.Ep)*

*%*

*% PEB.M.X- second-level (between-subject) design matrix*

*% PEB.M.W- second-level (within-subject) design matrix*

*% PEB.M.Q- precision [components] of second-level random effects*

*% PEB.M.pE- prior expectation of second-level parameters*

*% PEB.M.pC- prior covariance of second-level parameters*

*% PEB.M.hE- prior expectation of second-level log-precisions*

*% PEB.M.hC- prior covariance of second-level log-precisions*

*% PEB.Ep- posterior expectation of second-level parameters*

*% PEB.Eh- posterior expectation of second-level log-precisions*

*% PEB.Cp- posterior covariance of second-level parameters*

*% PEB.Ch- posterior covariance of second-level log-precisions*

*% PEB.Ce- expected covariance of second-level random effects*

*% PEB.F- free energy of second-level model*

*%*

*% DCM- first-level (reduced) DCM structures with empirical priors*

*%*

*% If DCM is an (N x M} array, hierarchical inversion will be*

*% applied to each model (i.e., each row) - and PEB will be a*

*% {1 x M} cell array.*

This routine inverts a hierarchical DCM using variational Laplace and Bayesian model reduction. In essence, it optimizes the empirical priors over the parameters of a set of first-level DCMs, using second-level or between-subject constraints specified in the design matrix *X*. This scheme is efficient in the sense that it does not require inversion of the first-level DCMs—it just requires the prior and posterior densities from each first-level DCM to compute empirical priors under the implicit hierarchical model. The output of this scheme (PEB) can be re-entered recursively to invert deep hierarchical models. Furthermore, BMC can be specified in terms of the empirical priors to perform BMC at the group level. Alternatively, subject-specific (first-level) posterior expectations can be used for classic inference in the usual way. Note that these (summary statistics) are optimal in the sense that they have been estimated under empirical (hierarchical) priors.

If called with a single DCM, there are no between-subject effects, and the design matrix is assumed to model mixtures of parameters at the first level. If called with a cell array, each column is assumed to contain first-level DCMs inverted under the same model.

### spm_dcm_loo

*% Leave-one-out cross-validation for empirical Bayes and DCM*

*% FORMAT [qE,qC,Q] = spm_dcm_loo(DCM,M,field)*

*%*

*% DCM - {N [x M]} structure DCM array of (M) DCMs from (N) subjects*

*% ——————————————————————*

*% DCM{i}.M.pE- prior expectation of parameters*

*% DCM{i}.M.pC- prior covariances of parameters*

*% DCM{i}.Ep- posterior expectations*

*% DCM{i}.Cp- posterior covariance*

*%*

*% M.X- second-level design matrix, where X(:,1) = ones(N,1) [default]*

*% field- parameter fields in DCM{i}.Ep to optimize [default: {'A','B'}]*

*%'All' will invoke all fields*

*%*

*% qE- posterior predictive expectation (group effect)*

*% qC- posterior predictive covariances (group effect)*

*% Q- posterior probability over unique levels of X(:,2)*

This routine uses the posterior predictive density over the coefficients of between-subject effects encoded by a design matrix *X*. It is assumed that the second column of *X* contains classification or predictor variables. A cross-validation scheme is used to estimate the mixture of parameters at the first (within-subject) level that are conserved over subjects in terms of a constant (first column of *X*) and differences (second column of *X*). Using a leave-one-out scheme, the predictive posterior density of the predictive variable is used to assess cross-validation accuracy. For multiple models, this procedure is repeated for each model in the columns of the DCM array.

## Synthesis

The decision was a result of the Reviewing Editor Gustavo Deco and the peer reviewers coming together and discussing their recommendations until a consensus was reached. A fact-based synthesis statement explaining their decision and outlining what is needed to prepare a revision is listed below. The following reviewers agreed to reveal their identity: Jean Daunizeau, Graham Murray

Synthesis of Reviews:

Significance Statement Comments for Author (Required):

This work has a strong potential for advancing the field, essentially because it lays down the practical route for researchers to use in their own research context sophisticated models and methods that have broad applicability potential.

Reviewer 1:

To begin with, I want to acknowledge that I already reviewed this manuscript when it was (unfortunately, unsuccessfully) submitted to another journal. I have updated my review as best as I could, but would like to apologize if authors feel that I am essentially making the same comments.

This is a proof-of-concept paper demonstrating the feasibility of a specific flavor of computational phenotyping from people's behaviour in instrumental learning tasks. In brief, authors exemplify a matlab toolbox (included in the SPM academic freeware) that can perform meta-Bayesian inference, i.e. Bayesian inference on Bayesian agents. They first simulate behavioural data from an instrumental learning task, and then fit computational models, eventually showing that model parameters capturing (virtual) inter-individual differences can be faithfully recovered. This is important, because this provides face validity with the authors' approach to computational phenotyping in psychiatry.

Overall, I found the paper well written, focused and methodologically sound. In addition, this is an important and timely endeavor, which is likely to be well received by the community. Nevertheless, I feel a few improvements are in order to be considered for publication in eNeuro.

First of all, authors adopt a rather subjective perspective on instrumental learning, which stems from their previous work on active inference. Although this is clearly not the main point of the paper, the resulting picture of computational phenotyping (as performed in the field) is debatable. This is illustrated by specific assertions such as: "This perspective suggests that the only things one can infer about a subject are their prior beliefs" (p.2) or "The preferred (desired) states are the states that the subject expects to find itself in. Therefore, a Bayes optimal subject will infer or select policies that bring about expected or preferred states." (p.6). These assertions are not facts, they are opinions, more precisely: authors' opinion that reward is an irrelevant concept that should be discarded and replaced with subjective priors about how to behave. I am sympathetic to this idea, and I do not challenge the fact that reward can be discarded: after all, authors' model predict instrumental behaviour without any reference to reward. However, from my point of view, there are two issues with such statements: (i) they do not convey a consensual view in the field (they may even be confusing), and (ii) they are detrimental to the main point of this paper (cf. last sentence of the abstract: "The procedural steps and routines we illustrate are not restricted to a specific field of research or particular computational model but can, in principle, be applied in many domains of computational psychiatry."). In fact, would authors be willing to tone down opinion statements (such as the above), they could highlight the fact that they actually provide the field with a formal way of comparing active inference with other computational models (e.g. reinforcement learning or Bayesian learning) given experimental data. On p. 14, they mention these other types of models that one could use to perform computational phenotyping ("[...]in practice researchers are also interested in comparing different models of the same (choice response) data. [...]"). But does the matlab toolbox described here actually deal with these classes of models? In turn, this would enable researchers to assess their relative predictive power and eventually evaluate their clinical utility (for, e.g., diagnostic purposes).

A second (and related) concern is that authors provide very little insight as to how one would actually derive a model for a given task. On p. 7, they write: "[...] the greatest challenge is to understand and define the hidden state space implicit in the paradigm [...]", to which I definitely agree. In fact, it might even be that the difference between, e.g., healthy controls and people suffering from psychiatric diseases might be best captured in terms of distinct hidden state spaces (an essential feature of the subjective model). The statistical framework proposed here solves the practical problem of estimating model parameters and/or comparing different models, but does not generate alternative cognitive scenarios in an automatic way. When describing their example, authors explain how they chose numerical values for priors/rewards in the simulated task, but do not justify the overall model structure (whose hierarchical form exploits the flexibility of variational Bayesian algorithms). Rather, they framed this choice as an authoritative opinion (e.g.: p. 8, "[...] we have to specify a hyperprior on precision. Precision reflects an agent's stochasticity or goal-directedness in choice behaviour but, crucially, itself has a Bayes-optimal solution that has to be inferred on a trial-by-trial basis"), as if there was no other alternative possibility. In fact, nowhere here is the overall model structure explicitly laid down, in a way that would enable the reader to both (i) identify its critical (and less critical) assumptions, and (ii) understand how the parameters enter the model. This is particularly problematic here because the reader is left with little guidance for discriminating between the assumptions that relate to the subjective model (the agent's priors) and those that relate to the objective model (the experimenter's priors). I would suggest that authors significantly improve this aspect of the manuscript, in the aim of educating potential users of the related SPM/DEM toolbox.

Lastly, I think the section on "inferring group effects" should be rewritten in the aim of clarifying the nature of the demonstration. For example, the manuscript does not contain enough information for readers to understand how difficult the issue of discriminating between (virtual) patients and controls is. Patients were simulated with subjective priors distinct from controls, and the proposed meta-Bayesian procedure proved able to recover this difference. But would a simple t-test on performance in the task have led to the same result (namely: a significant difference between the two groups)? If no, this is important, because this means that the meta-Bayesian approach can discriminate between different cognitive styles, to which simple performance analyses would be blind. If yes, then authors have to explain what the added-value of the model-based analysis is (given that a simpler diagnostic would be retrieved from performance differences). In the latter case, can authors change their simulation (such that the aggregate performance of controls and patients are matched), and evaluate the discriminative power of the proposed meta-Bayesian approach? This is critical for demonstrating the clinical utility of computational phenotyping. Another important point relates to the methodological strategy for inferring on the group membership. Authors could have chosen to rely on (Bayesian) model comparison, but, if I understand correctly, they did not: they used a cross-validation (leave-one-out) scheme. Could authors motivate this choice, and discuss the pros and cons of both approaches? Maybe authors could unpack the specific issues one is facing when trying to guess the group membership with computational phenotyping? And here again: can authors compare the predictive accuracy of their approach to that of a simple cross-validation scheme based upon mean performance in the simulated task? More generally, it seems to me that the main point of the manuscript would be significantly strengthened, would authors demonstrate with simulations that computational phenotyping is more sensitive than simple performance analyses (this may require more than one simulation)...

Note that I would anticipate that all these points would deserve a proper discussion in the last section of the manuscript, along with appropriate commentaries and comparisons w.r.t. existing alternative approaches. In other words, it would not be detrimental here to take some perspective about the paper's content, and replace it within the broader context of the field.

Reviewer 2:

This article provides background information relevant to a software tool the authors have developed, and describes the tool. The theory here is not new, although the paper provides a concise introduction and useful references for further reading. The strength of this paper is that is introduces the tool and it will most likely be cited by people who use the software tool.

I have some questions and suggestions

Line 62: please explain what a generative model is. This is explained later on but it should be explained here.

Line 280. What is a "shrinkage prior". Can these be altered in the tool? Could the authors be more specific than "fairly uninformative"?

Line 211

Precision is the inverse temperature of this distribution and itself is parameterised by a Gamma distribution with a rate or temperature (beta) hyperparameter. Thus, precision plays the same role as an inverse temperature in classical softmax choice rules, with the difference that it is continuously optimised. For our simulations we have set beta to a value of 2, resulting in an expected value for (inverse) precision of beta = 1.

Why do the authors switch between beta and symbol for beta?

Figures:

In Figure 4B, what do the red and blue colours mean in the top panel?

In Figure 4B, what do the different coloured dots mean in the second top panel?

Why is the y-axis labelled "expected utility" in 4B second top panel? What are the units of reaction times. How did the model estimate reaction times? No information is provided in the text about reaction times. These should be deleted from the manuscript or explained in more detail. Why are all the values negative?

Figure 7A "MAP appears in the figure but is not explained in the figure or the legend.

Figure 4B To equate precision with a catecholamine seems premature and over-simplistic. I think this will mislead users of the tool. The authors define precision mathematically, but it surely can't simply be equated with a molecule. In the brain there could be varying levels of this molecule in different regions, it could have different actions on different receptors, and according to tonic and phasic levels. My suggestion would be to cut the references to dopamine.

The authors use their model to simulate some data, utilising different parameters fro two putative groups of subjects (e.g. patients and controls). They then use their model to estimate a latent variable (beta) in each subject, and plot group means and group differences in figure 7B. Could the authors please plot what would (in a real experiment) be an observed, not latent variable: namely the number of rewards obtained (group mean and group difference effects).

Discussion: I think it would be helpful if the authors could say a bit more about the limitations of their theoretical framework and tool. When would the tool not be applicable or appropriate?
